# Physical Activity and Resilience among College Students: The Mediating Effects of Basic Psychological Needs

**DOI:** 10.3390/ijerph18073722

**Published:** 2021-04-02

**Authors:** Shanshan Xu, Zongyu Liu, Siyu Tian, Zhiyao Ma, Cunxian Jia, Guoxiao Sun

**Affiliations:** 1 School of Physical Education, Shandong University, Jinan 250061, China; xushanshan@mail.sdu.edu.cn (S.X.); 201700292037@mail.sdu.edu.cn (Z.L.); tiansiyu@mail.sdu.edu.cn (S.T.); mzyy@mail.sdu.edu.cn (Z.M.); 2 School of Public Health, Cheeloo College of Medicine, Shandong University, Jinan 250012, China; jiacunxian@sdu.edu.cn

**Keywords:** physical activity, resilience, autonomy need, competence need, relatedness need, college students, mental health

## Abstract

Considering the significance of resilience in coping with adversity, how to improve college students’ resilience is worthy of attention. Previous studies have revealed that physical activity can promote resilience; however, few studies examined the mediating factors between them. The purpose of this study was to investigate the effect of physical activity on resilience, as well as the mediating effects of competence need, autonomy need, and relatedness need between physical activity and resilience. The study involved 2375 college students (1110 males and 1265 females), with an average age of 20.25 years (*SD* = 2.04). Participants completed the International Physical Activity Questionnaire Short Form, Basic Needs Satisfaction in General Scale, and Connor-Davidson Resilience Scale. Results showed that physical activity was positively associated with resilience, and the three basic needs played significant mediating roles between physical activity and resilience. The indirect effect of competence need was significantly greater than that of autonomy need and relatedness need. To conclude, physical activity plays an important role in resilience among college students, and the satisfaction of competence, autonomy, and relatedness needs mediates the relationship between physical activity and resilience, among which, competence need appears as the strongest mediator.

## 1. Introduction

College students are confronted with a wide array of stressors that may lead to mental health issues, including academic challenges, relational adjustment, employment pressure, and some potential traumatic events [1]. Resilience is generally defined as the process of facing, overcoming, and growing from these stressors [2]. It is considered an important protective factor in mitigating the risk of stressful or adverse situations and promoting the healthy development of individuals [3,4,5]. Studies have shown that people with strong resilience are more psychologically healthy and less likely to have negative emotions [6,7,8,9], which indicates the importance of resilience. It enables people to thrive under challenging or threatening circumstances [10]. College students with high resilience tend to more actively mobilize internal and external resources to tackle the adversity, and they are less likely to have mental health problems, poor academic performance, and interpersonal problems compared to those with low resilience [11,12]. Given the great significance of resilience for college students, how to promote their resilience merits greater attention.

Evidence exists that physical activity is positively and significantly associated with resilience [13]. Secer and Yildizhan have suggested that the level of physical activity is an important variable that influences the psychological resilience level of university students [14]. Dunston et al. have indicated that physical activity contributes to the positive outcome of psychological resilience, and with the increase of vigorous physical activity, resilience is significantly enhanced [15]. San Román-Mata et al. have found that the more that college students participate in physical activity, the greater are their levels of resilience [16]. Xia et al. have suggested that physical activity is an effective way to foster college students’ resilience, which can improve their problem-solving ability, interpersonal communication ability, self-confidence, emotional regulation, and positive cognition [13].

Although some studies have revealed that physical activity is positively related to resilience [13,14,15,16], few studies have explored the mediating factors in the relationship between physical activity and resilience. Drawing from basic psychological needs theory may help us to understand the association between them. Basic psychological needs theory posits that competence need, autonomy need, and relatedness need are essential nutrients for human growth, integrity, and health [17,18]. Research has shown that when the basic needs are met, positive functioning and optimal mental well-being will follow [19,20]. Deci and Ryan have suggested that the three psychological needs function separately, and meeting each need is independently important [17]. Each basic need may have its own unique contribution in a specific domain. For example, Deci et al. have pointed out that there is a strong relationship between competence need satisfaction and job engagement [21]. Recent correlational and experimental evidence has revealed that the need for autonomy is the only mediator in the relationship between materialism and well-being [22]. Amato et al. have found that relatedness is the most important mediator as compared to competence and autonomy between total couple leisure satisfaction and marital satisfaction [23]. Therefore, we aimed to examine the effect of each psychological need in the physical activity-resilience link.

First, we propose that the satisfaction of competence need may be the mediating factor in the relationship between physical activity and resilience. Competence need refers to the perception that one is capable of achieving the desired goals [17]. It has been found to be significantly and positively correlated with resilience [24,25]. Neufeld and Malin have suggested that competence need satisfaction is associated with an increase in college students’ resilience and it likely represents a valuable avenue for supporting resilience [26]. In addition, physical activity has been supposed to be correlated with the satisfaction of competence need [27,28]. Babic et al. have reported a direct association between higher levels of physical activity and higher perceived competence [29]. By taking an active part in physical activities, college students’ perception of competence and self-efficacy may be satisfied. High perception of competence promotes positive expectations for achievement behaviors [30], which enables people to confidently and actively cope with the stress they encounter in the environment. In other words, their psychological resilience has been enhanced.

Second, the satisfaction of autonomy need may also play a mediating role between physical activity and resilience. Autonomy need is the belief that one’s actions are entirely self-chosen and controlled by one’s own will [17]. It demonstrates a positive correlation with resilience [31,32]. Martinek and Hellison have argued that the satisfaction of autonomy need is an effective way to acquire some degree of resilience for youth [33]. In addition, physical activity has been reported to have a positive effect on autonomy need satisfaction [27]. Fraguela-Vale et al. have demonstrated that engaging in physical activity, especially unstructured activity, is beneficial to the satisfaction of autonomy need [28]. Similarly, Doré et al. have suggested that physical activity could foster the perception of autonomy [34]. By actively participating in physical activities, college students may have a sense of self-determination and control over the environment, and their autonomy need will be satisfied. When college students feel free to make their own decisions, they may have greater adaptability and resilience to face and overcome the adversity they encounter.

Third, we believe that the satisfaction of relatedness need may be a mediator of the relationship between physical activity and resilience. Relatedness need is the belief that one is connected and valued by others [17]. It has been reported to be closely related to psychological resilience [24,31]. Rutter has suggested that when an individual develops positive and close relationships with others, a strong social support system will be created for dealing with adversity [35]. Perceived social support, respect, and understanding contribute to stronger psychological resilience that enables the individual to cope with stressors effectively [35]. Meanwhile, physical activity has been found to be positively associated with the satisfaction of relatedness need [27]. Stathi, Fox, and McKenna have revealed that physical activity allows people to interact socially with others and helps them build up a social support network [36]. Physical activity has also been reported to provide people with a greater sense of belonging, which satisfies their relatedness need [37]. Students may have close partners in physical activities and meet their need for relatedness. When they build up a good social network, they may have more courage and confidence to face pressure, setbacks, and difficulties.

**Figure 1:**
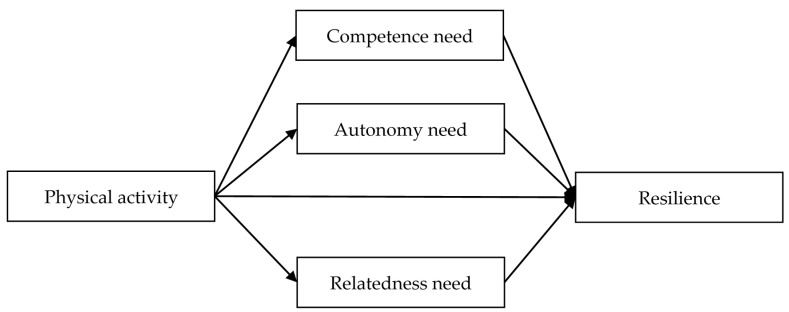
Structural framework.

To sum up, this study aims to explore the relationship between physical activity and resilience, as well as the mediating roles of competence need, autonomy need, and relatedness need between them. We expect that (1) physical activity is significantly and positively correlated with resilience in college students and (2) the relationship between physical activity and resilience is mediated by the satisfaction of competence need, autonomy need and relatedness need. The representation of the structural model is shown as follows (Figure 1).

## 2. Methods

### 2.1. Participants and Procedures

Participants in the present study were Chinese college students. We selected six universities (three in Shandong Province, one in Zhejiang Province, one in Sichuan Province, and one in Guizhou Province) as the sampling unit. We recruited participating students by the following ways: (1) advertising the questionnaire through social media; (2) printing out the link of the questionnaire and distributing it to the teaching buildings, departments, and dining rooms of students so that students could scan the link and fill in the questionnaire; and (3) students who had completed the questionnaire were encouraged to invite their classmates or friends to participate. Participants completed the questionnaire via the survey link. Before the survey, the purpose of this research and the voluntary nature of participation were clearly communicated to all the participants. They were informed that they could stop the survey at any time, and that completing the questionnaire indicated their informed consent to participate in this study. All data were collected on a voluntary, anonymous, and confidential basis. The Ethics Committee of the School of Public Health of Shandong University approved this study (No. 20190912).

### 2.2. Measures

#### 2.2.1. Demographic Factors

The demographic factors included gender, age, grade, major, residence, and whether the participant was an only child or not. Grades included freshmen, sophomore, junior, senior, and postgraduate. Majors were categorized as sciences, liberal arts, engineering, and medicine. Residence included urban or rural areas.

#### 2.2.2. The Short Form of the International Physical Activity Questionnaire (IPAQ-SF)

The Chinese version of the short from of the International Physical Activity Questionnaire (IPAQ-SF) was used to assess the physical activity of college students in this study [38]. The IPAQ-SF measures the number of days and the duration of each day of three types of activity: vigorous-intensity activities, moderate-intensity activities, and walking [39]. Each intensity of physical activity was weighted by its energy requirements in metabolic equivalent task (MET) minutes per week. A MET score was calculated by multiplying the MET value for each activity with duration (minutes) and frequency (days). Obtaining the amount of physical activity per week required the totaling of these three types of activities [39]. The IPAQ-SF has been proved to be a valid and reliable instrument for assessing physical activity levels and can be culturally adapted for the Chinese population [38].

#### 2.2.3. Basic Needs Satisfaction in General Scale (BNSG-S)

The Chinese version [40] of the Basic Needs Satisfaction in General Scale (BNSG-S) [41] was used to measure the fulfillment of psychological needs. The BNSG-S consists of 21 items, including 6 items measuring competence need, 7 items measuring autonomy need, and 8 items measuring relatedness need. The items were rated using a 7-point Likert scale from 1 “not at all” to 7 “very much”. In the whole scale, 12 items adopt positive scoring, such as “I feel like I can decide for myself how to live my life”, and the other 9 items adopt the reverse scoring method, such as “I often feel incompetent”. The higher the subscale score is, the greater the satisfaction of the corresponding need is. Cronbach’s alpha coefficient values for the competence subscale, autonomy subscale, and relatedness subscale were respectively 0.758, 0.705, and 0.827 in this study.

#### 2.2.4. Connor-Davidson Resilience Scale (CD-RISC)

The Connor-Davidson Resilience Scale (CD-RISC) was developed by American scholars Connor and Davidson [42]; it is a widely accepted measure of resilience [43]. In this study, the Chinese version of CD-RISC translated and modified by Yu and Zhang was used [44]. The CD-RISC is a 25-item self-report questionnaire with items rated on a 5-point Likert scale from 0 “never” to 4 “always”. The higher the CD-RISC scores, the greater the resilience. Cronbach’s alpha coefficient values for CD-RISC was 0.952 in the present study.

### 2.3. Statistical Analysis

The original data were exported from the Wenjuanxing questionnaire platform (https://www.wjx.cn/, accessed on 2 November 2020). SPSS 24.0 for Windows (IBM, Armonk, NY, USA) was used for statistical analysis. Descriptive statistics including frequency, mean, and standard deviation were tested to describe the distribution of demographic, basic psychological needs, and resilience variables. As physical activity variables were non-normally distributed, we applied the median and interquartile range to describe the distribution. The Spearman correlation was used to calculate the correlation between each pair of measures. Hayes’ PROCESS macro in SPSS (version 3.3) was performed in the mediation analysis [45]. The bootstrap method (sampling was repeated 5000 times) was used to estimate 95% confidence intervals (CIs) for significance testing of mediating effects. When the CI did not include zero, the direct or indirect effect was considered significant. All variables were standardized before entering the mediation model.

## 3. Results

### 3.1. Common Method Bias Analysis

We used factor analysis for common method bias testing. The chi-square statistic of Bartlett’s test of sphericity was significant (Kaiser-Meyer-Olkin (KMO) statistic = 0.973, *p* < 0.001). After analyzing the principal component, results revealed that the eigenvalues of 6 factors were >1 and the first factor explained 33.75% of the variance, which was lower than the threshold of 40%. This suggests the absence of serious common method biases in the present study [46].

### 3.2. Demographic Variables in the Sample

A total of 2954 questionnaires were commenced by students. After questionnaires with missing data were removed (n = 579), 2375 completed surveys were included for analysis. The response rate was 80.40%. The final sample consisted of 1110 (46.74%) male students and 1265 (53.26%) female students with a mean age of 20.25 years (*SD* = 2.04). Among the 2375 participating students, 1339 (56.38%) were from urban areas and 1103 (46.44%) were from one-child families. The distribution of students in each grade was: freshmen, 672 (28.29%); sophomore, 579 (24.38%); junior, 492 (20.72%); senior, 410 (17.26%), and postgraduate, 222 (9.35%). The distribution of students’ majors was as follows: sciences, 644 (27.12%); liberal arts, 757 (31.87%); engineering, 561 (23.62%); and medicine, 413 (17.39%).

### 3.3. Correlation Analysis

Descriptive statistics and a correlation matrix for physical activity, the satisfaction of three basic needs, and resilience are presented in Table 1. A bivariate correlation analysis showed that the variables were positively and significantly correlated with each other.

### 3.4. Regression Analysis

Table 2 demonstrates the regression coefficients of the mediating of the basic psychological needs of competence, autonomy, and relatedness between physical activity and resilience. The results showed that physical activity was significantly and positively associated with resilience and the satisfaction of three basic psychological needs. Meanwhile, the satisfaction of each basic need was positively associated with resilience.

### 3.5. Mediation Analysis

**Figure 2:**
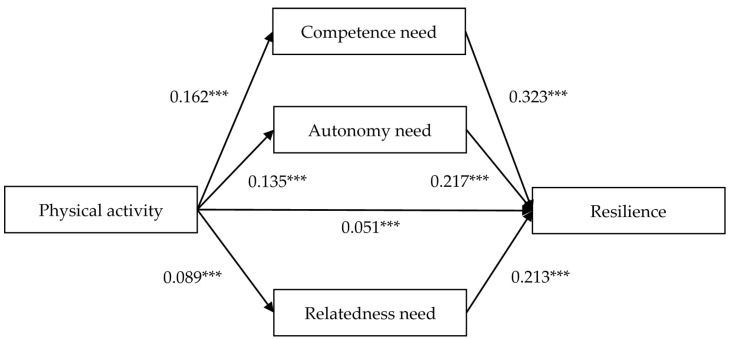
Mediation effect analysis of basic psychological needs satisfaction between physical activity and resilience; *** *p* < 0.001. Demographic variables were controlled as covariances.

Mediation analysis was carried out to estimate the indirect effects of physical activity on resilience that are mediated by competence need, autonomy need, and relatedness need. Table 3 and Figure 2 show the mediation analysis results. The indirect effects of physical activity on resilience via the mediation of competence need (95% CI: 0.036−0.069), autonomy need (95% CI: 0.018−0.042), and relatedness need (95% CI: 0.009−0.031) were all significant, which accounted for 34.44%, 19.21%, and 12.58% of the total effect, respectively. The differences of indirect effects between competence and autonomy and between competence and relatedness were 0.023 (95% CI: 0.007−0.039) and 0.033 (95% CI: 0.020−0.048), respectively. The CIs did not include zero, indicating that the mediating effect of competence need was significantly greater than that of autonomy need and relatedness need. The difference of the indirect effects between autonomy need and relatedness need was 0.010 (95% CI: −0.002−0.023), and the CI included zero, indicating that there was no significant difference between autonomy need and relatedness need. The results showed that competence need had the greatest mediating effect between physical activity and resilience.

## 4. Discussion

To our knowledge, this is the first study to examine the relationships between physical activity, basic psychological needs satisfaction, and resilience. Our major findings were as follows: (1) There was a significant and positive relationship between physical activity and resilience among Chinese college students; (2) the satisfaction of competence need, autonomy need, and relatedness need mediated the association between physical activity and resilience; and (3) the mediating effect of competence need was significantly greater than that of autonomy need and relatedness need.

Consistent with the previous studies [13,14,15,16], we found that physical activity was positively associated with resilience in our college student sample. The result is also supported by Hjemdal et al., who have argued that physical activity serves an important function in promoting psychological resilience [47]. Ozkara et al. have suggested that this may be because physical activity makes the cardiovascular system more efficient, and it does not need to do so much work to mobilize resources to cope with stressors [48]. Physical activity can also enhance the ability of emotional regulation by strengthening individual brain regions and large-scale neural circuits [49], so as to improve the mental resilience of college students. The more physical activity they engage in, the more resilient they will become [50]. In addition, Martinek and Hellison have pointed out that physical activity can stimulate optimism, hope, autonomy, and social support, which are core attributes of resilience [33]. In other words, physical activity may provide an appropriate setting for the acquisition of resilience attributes. Given that college students must endure many pressures and challenges in their study and daily life, greater involvement in physical activity is important, and it may represent a valuable way to support their resilience.

The present study found that the fulfillment of the competence need had the greatest mediating effect on the relationship between physical activity and resilience among college students. Our finding supports previous studies that physical activity exhibited a positive association with the degree of satisfaction of competence need [27,28]. A similar outcome was reached by Wilson et al., who indicated that people reported high levels of competence need satisfaction in exercise [51]. According to Ryan and Deci, the satisfaction of competence need occurs in high-quality social experiences [52]. Engaging in physical activity may acquire these social experiences, such as cooperation, obtaining feedback, and supporting the success of each other, which help to meet the participants’ needs for competence [34]. In line with the previous research [26], we found that competence need satisfaction was positively correlated with psychological resilience. To demonstrate greater resilience, individuals need to expend a lot of cognitive and behavioral effort [53]. According to basic psychological needs theory, the satisfaction of competence need enables people to maintain a high level of effort in the face of adversity [17]. In other words, competence need satisfaction promotes the perception of self-efficacy that results in the maintenance of high levels of effort-behaviors that reflect greater psychological resilience.

As shown by the results, the satisfaction of autonomy need exerted a small (nearly 20%) but significant mediating effect on the relationship between physical activity and resilience. We found that physical activity was positively associated with the satisfaction of autonomy need, which is consistent with previous studies [54,55,56]. Physical activity may be an antecedent to autonomy need satisfaction [55]. This is perhaps because physical activity can create an autonomy-supportive environment in which individuals feel a sense of self-determination. When individuals view their behaviors as self-endorsed, their need for autonomy is fulfilled [17]. People whose autonomy needs are satisfied are more likely to evaluate the situation as challenging rather than threatening, and have a higher perception of control over the environment [57]. This highlights how autonomy need satisfaction helps people cope with adversity and develop the psychological resilience required.

This study also revealed that the mediating effect of relatedness need satisfaction between physical activity and resilience is minimal (12.58%) but different from zero. The relatedness need is satisfied when individuals feel that they are significant and loved [17]. Physical activity can provide a context for social interaction where people foster connections with others and feel accepted [34]. Furthermore, the satisfaction of relatedness need promotes the perception of self-value and importance, which may enable the individual to cope with adversity or pursue goals with sustained efforts. That is, psychological resilience has been improved.

We found that the mediating effect of competence need between physical activity and resilience was significantly greater than that of autonomy need and relatedness need. This result supports the findings of Taylor et al., who have found that physical activity is more closely related to the satisfaction of competence need than to autonomy and relatedness needs [58]. This result also supports the study of Neufeld and Malin, who have suggested that resilience has the greatest correlation with competence compared with autonomy and relatedness, and only competence was associated with well-being through the mediating effect of resilience [26]. Taken together, the above results reveal the significance of considering the role of meeting competence need in the relationship between physical activity and resilience.

This study has significant implications. First, we provide evidence for the importance of physical activity in improving college students’ psychological resilience. Identifying the influencing factor (i.e., physical activity) would help to formulate corresponding strategies to promote resilience, which is helpful for college students to cope with threatening conditions and improve their well-being. Moreover, we apply the basic psychological needs theory to explain the association between physical activity and resilience, and therefore provide a theoretical basis for understanding the underlying mechanism. From this perspective, more effective and feasible physical activity programs can be designed in the future to satisfy autonomy need, relatedness need, and especially competence need of college students and thus improve their resilience.

Despite these positive findings, the current study is not without limitations. First, this is a cross-sectional study, therefore, the causal relationship between variables cannot be established. A longitudinal design should be employed in future research. Second, we used the self-reported scale IPAQ-SF to measure physical activity. Further research should consider using more objective measuring devices (e.g., accelerometers, pedometers) to measure the amount of physical activity. Third, participants were college students, and therefore the results of this sample may not be extended to other populations. Fourth, we suggest future research consider other variables that might influence the relationships in the study, for instance, internal locus of control, coping style, and so on.

## 5. Conclusions

This study demonstrates that physical activity is significantly and positively associated with resilience among college students. More importantly, competence need, autonomy need, and relatedness need play mediating roles between physical activity and resilience, and competence need appears as the strongest mediator. We suggest that actively participating in physical activities to satisfy the basic psychological needs, especially competence need, might help to improve college students’ resilience.

## Figures and Tables

**Table 1:** Descriptive statistics and correlation matrix for physical activity, basic psychological needs satisfaction, and resilience.

Variables	Physical Activity	Competence Need	Autonomy Need	Relatedness Need	Resilience
Physical activity	1.000	-	-	-	-
Competence need	0.112 **	1.000	-	-	-
Autonomy need	0.101 **	0.675 **	1.000	-	-
Relatedness need	0.045 *	0.680 **	0.686 **	1.000	-
Resilience	0.159 **	0.605 **	0.598 **	0.586 **	1.000
M/Me	1908.000	27.099	31.343	39.534	90.067
SD/IQR	2167.000	5.532	5.668	7.463	15.227

Notes: M = mean; SD = standard deviation; Me = Median; IQR = Interquartile Range; * *p* < 0.05, ** *p* < 0.01.

**Table 2:** Regression coefficients of the mediating of basic psychological needs satisfaction between physical activity and resilience.

Outcome Variables		Goodness-of-Fit Indices			Regression Coefficient and Significance	
	Predictors	*R*	*R* ^2^	*F*	*Β*	*t*
Resilience		0.223	0.050	17.686 ***		
	Physical activity				0.151	7.398 ***
Competence need		0.209	0.044	15.366 ***		
	Physical activity				0.162	7.887 ***
Autonomy need		0.188	0.035	12.353 ***		
	Physical activity				0.135	6.552 ***
Relatedness need		0.189	0.036	12.517 ***		
	Physical activity				0.089	4.309 ***
Resilience		0.704	0.496	232.535 ***		
	Physical activity				0.051	3.363 ***
	Competence need				0.323	13.501 ***
	Autonomy need				0.217	8.993 ***
	Relatedness need				0.213	9.168 ***

Notes: *** *p* < 0.001. Demographic variables were controlled as covariances.

**Table 3:** Mediating effects of basic psychological needs satisfaction between physical activity and resilience by the PROCESS macro. Demographic variables were controlled as covariances.

Effect Types	Path	95% CI	Effect
Direct effect	Physical activity → Resilience	0.021−0.081	0.051
Indirect effect	Physical activity → Competence need → Resilience	0.036−0.069	0.052
	Physical activity → Autonomy need → Resilience	0.018−0.042	0.029
	Physical activity → Relatedness need → Resilience	0.009−0.031	0.019
Total indirect effect	-	0.068−0.133	0.100
Total effect	-	0.111−0.191	0.151
(C1)	-	0.007−0.039	0.023
(C2)	-	0.019−0.048	0.033
(C3)	-	−0.002−0.023	0.010

Notes: CI: confidence interval. Specific indirect effect contrast definitions: (C1) = competence need minus autonomy need; (C2) = competence need minus relatedness need; (C3) = autonomy need minus relatedness need.

## Data Availability

The data presented are available on request from the corresponding author.
